# Body Mass Index Did Not Affect the Risk of Revision 3-9 Years After Total Knee Replacement Surgery

**DOI:** 10.1016/j.artd.2024.101376

**Published:** 2024-04-16

**Authors:** Jan Rune Mikaelsen, Rune Bruhn Jakobsen, Jan Harald Røtterud, Per-Henrik Randsborg

**Affiliations:** a Department of Orthopaedic Surgery, Akershus University Hospital, Lørenskog, Norway; b Faculty of Medicine, Institute of Clinical Medicine, Campus Ahus, University of Oslo, Lørenskog, Norway; c Faculty of Medicine, Institute of Health and Society, University of Oslo, Oslo, Norway

**Keywords:** Total knee replacement, Obesity, BMI, Risk of revision

## Abstract

**Background:**

There are conflicting reports in the literature regarding the risk of revision after primary total knee replacement (TKR) in obese patients. The purpose of this study was to investigate if body mass index (BMI) influences the risk of revision 3-9 years after primary TKR.

**Methods:**

All patients undergoing a primary TKR in our institution from 2014 to 2018 were included in a retrospective study. The effect of BMI on all-cause revision was estimated in a logistic regression analysis. A directed acyclic graph was created to identify variables affecting the primary endpoint (revision). According to the directed acyclic graph, adjustment was only needed for age and smoking. However, we also included variables thought to influence the revision risk based on clinical experience and previous research. The final logistic regression analysis was therefore adjusted for age, sex, smoking status, diabetes mellitus and the American Society of Anesthesiologists classification.

**Results:**

One thousand fifty-nine primary TKR patients with a mean age of 68.1 (standard deviation 9.4) years were included. There were 609 (57.5%) women, and the median follow-up time was 5.6 (range 3.0-9.0) years. There were 41 (3.9%) revisions. BMI did not affect the risk of revision when adjusted for relevant covariates in a multivariate logistic regression analysis (odds ratio 0.99, 95% confidence interval 0.93-1.05, *P* = .6).

**Conclusions:**

BMI did not influence the risk of revision rate 3-9 years after TKR.

## Introduction

The prevalence of overweight and obesity has increased in the last decades and, according to the World Health Organization, has become a serious health concern globally [1]. Obesity is a well-documented risk factor for the development of osteoarthritis (OA) [[2], [3], [4]]. The increasing prevalence of obesity is predicted to accelerate the demand and need for total knee replacement (TKR) [[5], [6], [7]]. Results from several studies suggest that more than 30% of all patients receiving total hip replacement and TKR are obese [[7], [8], [9], [10], [11]]. A weight gain of 5 kg increases the risk of knee OA by 30% [12]. The anticipated increase in revision TKRs may pose major problems for the health care system. Optimization of common modifiable risk factors prior to elective primary TKR, such as smoking, diabetes mellitus, poor dentition, and obesity, may ultimately help decrease rates of revision surgery [13].

The success, failure, and clinical outcome after TKR may potentially be diverging in obese patients compared to nonobese patients [[14], [15], [16], [17]]. There are conflicting reports on the relationship between obesity and clinical outcomes following TKR. Some authors report that obese patients have a poorer functional outcome and a higher revision rate after TKR [18,19], whereas other studies report equivalent results between obese and nonobese patients following TKR [20,21]. In addition, the paper from Chen JY et al. concludes that obese patients have better functional improvement following TKR than nonobese patients [22]. A systematic review from 2016 aimed to assess the existing literature on the safety, outcomes, and complications associated with TKR in obese patients and concluded that more studies are needed [23].

The aim of the study was to investigate the effect of obesity on the risk of revision after primary TKR. We hypothesized that there is no significant effect of obesity on the risk for revision after TKR.

## Material and methods

The primary research question was to determine whether the risk of revision of TKR was influenced by body mass index (BMI). The primary endpoint was revision of the TKR for any reason. Revision was defined as the removal, addition, partial, or total exchange of prosthetic implants for any reason.

**Figure 1:**
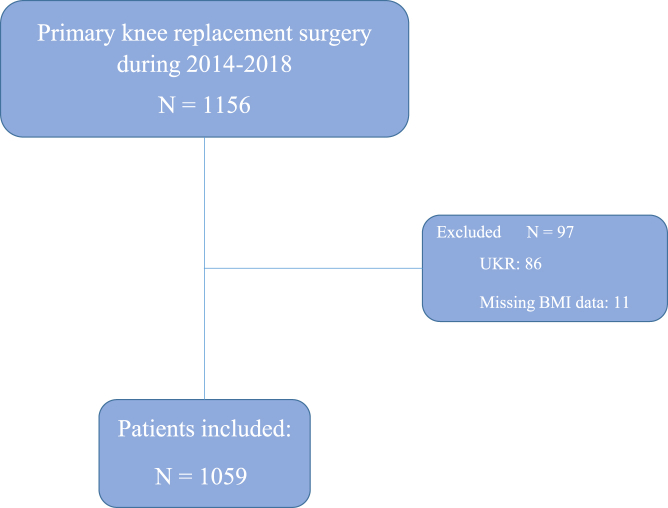
Flow chart of included patients. UKR, unicompartmental knee replacement.

This study was conducted at Akershus University Hospital (Ahus), Norway, between January 2014 and December 2018 (Fig. 1). Patients who underwent a primary TKR were included. All patients received a cemented NexGen CR/PS total knee prosthesis (Zimmer Biomet, Warszawa, IN) via a standard medial parapatellar approach to the knee joint. The patients were identified through a search of the procedure codes in the electronic patient journal system. The relevant clinical data were extracted, such as sex, age at primary surgery, height, weight, comorbidities, and complications. The treatment of major complications such as deep infections, periprosthetic fractures, and revisions are systematically recorded in the Norwegian Arthroplasty Register. The included patients were subsequently cross-checked with Norwegian Arthroplasty Register to identify any patients receiving revision surgery at a different hospital during the follow-up. The patients were followed up for a minimum of 3 years.

The exposure of interest was BMI. BMI is the most common tool used to grade obesity. We evaluated BMI in 5 categories according to the World Health Organization’s classification, normal weight (BMI <24.9 kg/m^2^), overweight (BMI 25-29.9 kg/m^2^), obese class I (BMI 30-34.9 kg/m^2^), obese class II (BMI 35-39.9 kg/m^2^), and obese class III (BMI >40 kg/m^2^). The following patient variables were assessed at the time of surgery and used to perform adjusted analysis: BMI, sex, age, American Society of Anesthesiologists (ASA) Physical Status Classification, smoker/nonsmoker, and diabetes yes/no.

The study was planned and executed according to the tenets of the World Medical Association Declaration of Helsinki [24]. The participants provided written informed consent before inclusion in the arthroplasty register. The project was approved by the Regional Committee for Medical Research Ethics—South East Norway (REK 2019/701 A) and the data protection officer of the institution (PVO 2019_44).

### Statistical analysis

**Figure 2:**
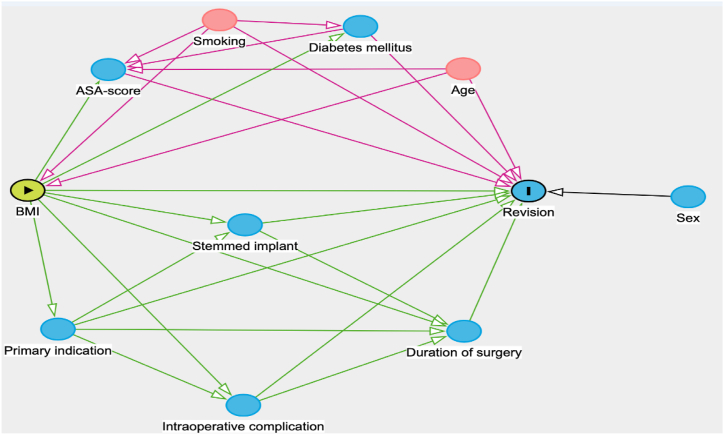
DAG visualizing the effect of BMI on revision through observed variables. Exposure, outcome, ancestor of outcome, ancestor of outcome and exposure (confounder). = exposure = outcome = ancestor of outcome = ancestor of outcome and exposure (confounder)

Logistic regression analysis was performed to estimate the effect of BMI on the 3-9-year revision risk after TKR. To guide the selection of variables in the regression analysis, we constructed a directed acyclic graph (DAG) using DAGitty [25] (Fig. 2). DAGs illustrate concepts such as confounding, selection bias, and the distinction between total, direct, and indirect effects [26]. The DAG visualized that only adjustment for age and smoking status was needed in the model to estimate the total effect of BMI on revision risk.

A traditional logistic regression analysis was also performed, estimating the effect of BMI on the 3-9-year revision risk after TKR, adjusted for age, sex (ref: female), smoking status (ref: nonsmoker), diabetes (ref: nondiabetic), and ASA classification (dichotomized to reference group: ASA 1 or 2).

Statistics were presented as odds ratios and 95% confidence intervals. Descriptive statistics were presented as mean and standard deviation for continuous variables. A *P*-value <.05 was considered to indicate statistical significance. Statistical analysis was performed using SPSS 22 (IBM Corporation, Armonk, NY).

## Results

Of the 1070 primary TKRs corresponding to the inclusion criteria, BMI information was missing in 11 (1.02%), leaving 1059 TKRs for inclusion, of which 609 (57.5%) were women. The mean age at the time of surgery was 59.1 years. The median follow-up time was 5.6 (range 3.0-9.0) years. There were 41 (3.9%) revisions during the follow-up period, of which 14 (34%) patients were revised due to infection, 14 (34%) patients were revised due to instability or malalignment, 5 (12%) patients were revised due to aseptic loosening, and 8 (19.5%) patients were revised due to pain or other nonspecified reasons. The majority of patients (64%) were overweight (BMI 25-30) or moderately obese (BMI 30-35) (Table 1). Fifty-nine (5.6%) patients were very severely obese (BMI>40), of which eleven had a BMI >45. Of these eleven patients, there was one revision.

**Table 1:** Patient characteristics.

Characteristic	Total	Normal weight	Overweight	Moderately obese	Severely obese	Very severely obese
BMI		18.5-25	25-30	30-35	35-40	>40
n	1059	191	357	325	127	59
Age, mean (SD)	68.1 (9.4)	70.4 (10.5)	69.4 (8.9)	67.1 (9.2)	65.5 (8.7)	64.3 (8.0)
Females, n (%)	609 (57.5)	112 (58.6)	184 (51.5)	184 (56.6)	91 (71.7)	38 (64.4)
BMI, mean (SD)	30.2 (5.5)	23.1 (1.56)	27.6 (1.4)	32.2 (1.4)	36.7 (1.4)	43.1 (4.8)
Ever smokers, n (%)	370 (34.9)	76 (39.8)	123 (52.5)	109 (33.5)	43 (33.9)	19 (32.2)
Diabetes, n (SD)	140 (13.2)	15 (7.8)	38 (11.9)	59 (18.2)	17 (13.4)	11 (18.6)
ASA^a^ 3-4, n (%)	223 (21.2)	32 (16.9)	59 (16.7)	59 (18.3)	30 (23.6)	43 (74.1)
Revisions, n (%)	41 (3.9)	7 (3.6)	14 (3.9)	16 (4.9)	1 (0.8)	3 (5.1)

SD, standard deviation.

a Missing data for 9 patients (2 in normal weight, 3 in overweight, 3 in moderate, and 1 in very severely obese).

BMI did not influence the 3-9-year revision risk after TKR, according to the logistic regression analysis adjusted for age and smoking status (Table 2). The effect of BMI on revision risk remained nonsignificant when adjusting for more traditional explanatory variables such as sex, ASA classification 3-4, and diabetes. Age had a significant effect on revision risk, with younger patients at higher risk (odds ratio 0.97, 95% confidence interval 0.93-0.99, *P* = .04) (Table 3).

**Table 2:** Logistic regression analysis.

Variable	OR	95% CI	*P*-value
BMI	0.99	0.93-1.05	.6
Smoker	0.90	0.46-1.74	.7
Age	0.97	0.94-1.00	.07

OR, odds ratio; CI, confidence interval.

The effect of BMI on the 3-9-year revision risk after primary total knee replacement. Estimates adjusted for age and smoking status (data presented).

**Table 3:** Logistic regression analysis.

Variable	OR	95% CI	*P*-value
BMI	0.97	0.92-1.03	.4
Age	0.97	0.93-0.99	.04
Sex	1.11	0.58-2.13	.7
Smoker	0.90	0.46-1.77	.8
Diabetes	1.98	0.90-4.35	.09
ASA 3-4	1.34	0.61-2.93	.5

OR, odds ratio; CI, confidence interval.

The effect of BMI on the 3-9-year revision risk after primary total knee replacement.

Estimates adjusted for age, sex (ref: female), smoking status (ref: nonsmoker), diabetes (ref: nondiabetic), and ASA classification (dichotomized to reference group ASA 1 or 2).

## Discussion

We found no statistically significant higher risk of revision after TKR in any of the BMI classification groups. Our results are in line with previous studies that have found no correlation between obesity and the risk of revision [27,28]. The literature was recently aggregated in a meta-analysis that showed that a higher BMI did not increase revision rates after unicompartemental knee replacements, and therefore obese patients should not be denied surgery based on BMI alone [28]. However, a high-quality meta-analysis of 20 studies (15.276 patients) found a higher revision rate in obese (BMI over 30) patients undergoing TKR [17]. A retrospective cohort study of 2442 primary TKR found that the risk of revision was doubled in patients with a BMI over 35 kg/m^2^ [29]. These conflicting results indicate that there is a complex relationship between obesity and the risk of adverse outcomes following TKR. This is demonstrated by the Danish arthroplasty register, which found a complex association between patient weight and knee arthroplasty survival, with an increased risk of revision in patients older than 70 years with a weight either under 60 kg or over 80 kg [30]. Weight was not found to affect the risk of revision in patients aged 55-70 years. However, there was no possibility to investigate the correlation between BMI and revision rate, as the patients’ height was not available. The ability of BMI to reflect body habitus is limited. Therefore, the variables weight and height are suggested to be individual predictors of risk [[31], [32], [33]]. A report from the Swedish Knee Arthroplasty Register showed that weight and height were better predictors of risk of revision individually than combined with BMI [34]. In our study, height, weight, and BMI were registered in all patients, but only age influenced the risk of a revision.

Some hospitals offer TKR to patients without weight loss, while other hospitals advise obese patients to undergo weight loss before considering TKR. Weight loss is often difficult to achieve for patients with knee OA due to the body habitus, pain, and stiffness of the knee OA [35] and there is often an assumption by patients that weight loss will occur once their pain is relieved by TKR [36]. A recent meta-analysis could not demonstrate a clear relationship between weight loss prior to TKR and reduction in complications [37]. Studies that have found a benefit of weight loss for knee OA have not included patients with a BMI above 40 kg/m^2^ or more advanced knee OA. Furthermore, there is unclear evidence of a benefit of presurgical weight loss on TKR outcomes. A randomized controlled trial showed a significant reduction in complications in patients with a BMI greater than or equal to 35 who underwent weight loss surgery before TKR surgery [38]. Another study found that the sequence of surgery in patients undergoing both weight loss surgery and TKR did not influence the revision risk [39]. However, the above-mentioned studies included patients younger than 65 years, which means that the majority of TKR patients are not represented. In our study, the mean age was 68 years, and we found that younger age is an independent risk factor for revision. These are important evidence gaps, suggesting that recommendations for BMI reduction prior to TKR should be tempered by the current uncertainty in the literature.

One of the factors contributing to lower implant survival in obese TKR patients is aseptic loosening, particularly in relation to the tibial component [40,41]. One solution to this is the use of tibial stems to improve fixation and more evenly distribute biomechanical forces on the bone/implant interface in these patients [42]. However, a newly published national registry study from Australia compared the reason for revision, rate, and type of revision between primary TKR using stemmed tibial prostheses, stratified by BMI and obesity. They found no statistically significant difference in the rate of revision for loosening with or without the use of tibial stems when stratified by BMI [42].

Revision TKRs are associated with a higher complication and reoperation rate when compared to primary TKRs [43]. Bigham et al. showed that obese and morbidly obese patients showed an increased risk of rerevision (1.6- and 1.7-times, respectively) in comparison to normal-weight patients [44]. However, it is important to acknowledge that morbidly obese patients have been shown to benefit equally to nonobese patients in terms of functional outcomes following revision TKR, despite their increased risk of complications [45].

Our results can help target treatment optimization to the group most in need and avoid labeling patients at high risk when, in fact, they are not [46].

## Implications

Our results show that obese people with knee OA do not have a higher risk of all-cause revision after TKR compared to nonobese people. Surgeons should be careful not to undertreat obese patients under the misconception that they may have a higher revision risk. The strongest predictor for revision was younger age, not BMI. However, we suggest more prospective studies to understand the importance of BMI and revision risks when undergoing TKR surgery.

## Limitations

This study has several limitations. First, this was a retrospective study, and the patients were not specifically recalled for clinical or radiological review. Revision is not the only outcome of interest following TKR. However, the national register does not collect other possible adverse or unwanted effects, such as medical complications and inferior functional results, so the effect of BMI on these outcomes could not be estimated in our study. The patients were operated at a single institution with a relatively homogenous population, which may limit the external validity of the results. However, the procedures were performed by several orthopaedic surgeons, which is often seen as an advantage.

The follow-up time of 3-9 years is relatively short, and the revision risk may differ between BMI groups later. Our cohort was relatively small, with only 41 revisions, making firm conclusions difficult to draw with the possibility of a type II error. The retrospective nature of the study is also a limitation, although this yields real-world data from a typical hospital setting, which may be regarded as a strength. We had no direct measurement of activity level on patient-reported outcome. Our statistical model was adjusted for age, sex, BMI, smoker, and ASA score, which have been shown to correlate well with patient activity, physical activity before and after primary hip [47].

## Conclusions

In this study, we found no statistically significant higher risk of revision between nonobese and obese patients undergoing primary TKR. Age, on the other hand, affects the revision risk, with younger patients being at higher risk. Obesity did not represent a clear risk factor for failure and should not be considered a definite contraindication for a TKR.

## Acknowledgments

The authors would like to thank Anne Marie Fenstad and Ove Furnes at the Norwegian Arthroplasty Registry for collecting revision data from other institutions.

## Funding

The study was funded by the 10.13039/501100006095South-Eastern Norway Regional Health Authority (grant number 2023088).

## Conflicts of interest

J.H. Røtterud is an editorial board member of the Orthopaedic Journal of Sports Medicine. P.-H. Randsborg receives royalties from Universitetsforlaget for the book “Brudd og Skadebehandling, en metodebok,” a medical text book on fracture management (unrelated to the topic of this paper), is an associate editor for the Journal of Bone and Joint Surgery Open Access, is an editorial board member for the Journal of the Norwegian Medical Association, and is President of the Norwegian Orthopaedic Association (2024-2025). All other authors declare no potential conflicts of interest.

For full disclosure statements refer to https://doi.org/10.1016/j.artd.2024.101376.

## CRediT authorship contribution statement

**Jan Rune Mikaelsen:** Writing – original draft, Methodology, Formal analysis, Data curation. **Rune Bruhn Jakobsen:** Writing – review & editing, Supervision, Methodology, Formal analysis. **Jan Harald Røtterud:** Writing – review & editing, Supervision, Methodology, Conceptualization. **Per-Henrik Randsborg:** Writing – review & editing, Supervision, Project administration, Funding acquisition, Formal analysis, Conceptualization.

## Appendix A Supplementary Data

Conflict of Interest Statement for Jakobsen

Conflict of Interest Statement for Mikaelsen

Conflict of Interest Statement for Randsborg

Conflict of Interest Statement for Røtterud
